# Age dependent prevalence of the supraacetabular fossa in children, adolescents and young adults

**DOI:** 10.1186/s13244-022-01229-0

**Published:** 2022-05-13

**Authors:** Desiree Vaeth, Tobias Johannes Dietrich, Simon Wildermuth, Sebastian Leschka, Stephan Waelti, Nicole Graf, Tim Fischer

**Affiliations:** 1 Division of Radiology and Nuclear Medicine, Cantonal Hospital St. Gallen, Rorschacher Strasse 95, 9007 St. Gallen, Switzerland; 2 Faculty of Medicine, University of Zurich, Pestalozzistrasse 3, 8091 Zurich, Switzerland; 3 Clinical Trials Unit, Cantonal Hospital St. Gallen, Rorschacher Strasse 95, 9007 St. Gallen, Switzerland

**Keywords:** Magnetic resonance imaging, Hip, Pediatrics

## Abstract

**Objectives:**

The supraacetabular fossa (SAF) is an anatomical variant of the acetabular roof which may mimic a cartilage defect. Two different subtypes have been described: type 1 fluid-filled and type 2 cartilage-filled. The adult prevalence of SAF was reported between 10.5 and 12.6%. We aimed to determine SAF prevalence in a pediatric and young adult population and examine the potential remodeling of the subtypes over time.

**Methods:**

A retrospective search of the institutional database for hip MRI of participants aged 4–25 years was carried out between 2010 and 2020. A total of 401 eligible MRIs of 323 participants were analyzed by two readers. The documented features were: existence of SAF, definition of subtype and measurements of the SAF in three dimensions. Logistic regression models were calculated to estimate the influence of age on the presence of SAF.

**Results:**

Out of 323, 115 (35,6%) participants demonstrated a supraacetabular fossa. 63 (19.5%) participants presented subtype 1 and 51 (15.8%) type 2; one participant had both. The predicted probability for SAF increases until the age of 14, beyond 14 years, the combined predicted probability for both subtypes decreased again. In contrast to SAF type 1, SAF type 2 was more prevalent with older age. The size of the SAF decreased with aging.

**Conclusion:**

The supraacetabular fossa is most frequent in adolescents. With higher age, the prevalence and the size of the SAF decreased. This data supports the theory that the SAF is a developmental variant.

## Key points

1. Supraacetabular fossa (SAF) is most frequently observed at approximately 12–17 years of age.
2. With higher age, the prevalence and the size of the SAF decreased.
3. The present data support the theory that the SAF is a developmental variant.

## Introduction

Nowadays, MR imaging of the hip is one of the most commonly performed imaging techniques in musculoskeletal radiology to arrive at a wide range of differential diagnoses such as fractures, labral tears, cartilage defects and injuries of muscles and tendons [1]. Due to multiple anatomic structures and complex biomechanics of the hip, image interpretation is challenging. Especially in a pediatric population, in which hip pain and limping are common complaints [2, 3] and who are difficult to evaluate clinically, the awareness of developmental variants of the hip joint is indispensable to establish the correct diagnosis. Common variants include synovial folds [4] or a sublabral recess [5]. The supraacetabular fossa (SAF), a pseudodefect of the acetabular cartilage is one of these variants that can mimic pathology [6]. In a pediatric population, the pathologies which the SAF can be misinterpreted for are cartilage defects after septic arthritis or osteomyelitis of the pelvis with spreading to the hip joint. Another pathology that reveals a similar picture as a SAF could be a traumatic pelvic fracture with involvement of the acetabulum. The SAF was first described by Byrd in 2006 [7]. Subsequently, the imaging characteristics of the SAF were reported in 2012 [8] and 2018 [9]. The SAF is a well-circumscribed bony fossa in the acetabular roof near the 12 o'clock position [7, 8]. On MR images, two subtypes are distinguished; type 1 is filled with synovial fluid or contrast agent and type 2 is filled with cartilage [7, 8]. The prevalence of the SAF was 10.5% in a study population older than 16 years [8] and 12.6% in a population with a mean age of 35.9 years [9]. It was hypothesized that the SAF is a developmental variation and undergoes an age-dependent remodeling over time [8, 10]. To our knowledge, the age-dependent prevalence of the SAF in children, adolescents and young adults has not been reported so far in the peer-reviewed literature.

The primary aim of this study was to determine the age-dependent prevalence of the SAF in children, adolescents and young adults since there is not yet an evidence-based consensus regarding its etiology. The hypothesis that the SAF may be a developmental variant was proposed but never proven in a younger population. Data derived from this study is clinically relevant to familiarize radiologists with this specific entity and thereby avoid pitfalls regarding the above-mentioned mimickers of a SAF in a pediatric population but also allows more confidence when encountered in an adult population regarding the developmental nature of the SAF.

The secondary aim was the evaluation of sensitivity and specificity of radiography for detection of a SAF with MRI taken as gold standard.

## Materials and methods

### Participant inclusion

Institutional board approval was obtained. A retrospective review of our institutional digital database (St. Gallen Cantonal Hospital and Children’s Hospital of Eastern Switzerland) was carried out for MRI examinations of the hip (MR arthrography, without contrast agent and with i.v. contrast agent) in the period between 2010 and 2020. Participants between 4 and 25 years of age were included. The upper age limit was set to 25 years based on the forensic age diagnostic knowledge, that around this age, the last plate of the collarbone closes and especially the ossification of the hip has finished between 20 and 25 years [11, 12].

Due to the lack of an example of the SAF in newborns and infants in the current literature, we screened all infants and young participants for the presence of a SAF. The youngest participant with a clearly visible SAF as described by Dietrich et al. [8] had an age of four, and we concluded that a SAF -if present- could be identified at the age of four years and older. The lower age limit is hence set to four years. All participants without informed consent were excluded. Disorders, directly involving the bone and cartilage of the hip joints were excluded, in specific: hip dysplasia, capital femoral epiphysis, Perthes disease, inflammatory arthritis and degenerative diseases. MRIs with severe motion artifacts were also excluded. The primary retrospective review of our institutional digital database for MR-imaging of the hip revealed 422 possible eligible participants with 492 MR images of hip joints. MR images were excluded for the following reasons: Underlying pathology of the hip (*n* = 69; hip dysplasia *n* = 59, capital femoral epiphysis *n* = 2, Perthes disease *n* = 5, inflammatory arthritis *n* = 1, degenerative diseases *n* = 2), no informed consent (*n* = 5), poor imaging quality (*n* = 4).

### Imaging

All participants were scanned using 1.5-Tesla or 3-Tesla MRI scanner (Magnetom Skyra, Magnetom Skyrafit, Magnetom Vida, Magnetom Avantofit and Magnetom Aera, Siemens Healthineers, Erlangen, Germany). Mainly standardized protocols for scans without contrast agent, with contrast agent and MR-arthrography were used but sometimes differed due to the various indications for imaging, variation in age of participants and changes of our image acquisition over time. As an example, the protocol for MR-arthrography on a 3-Tesla Skyra scanner is given in Table 1. Most participants underwent either proton density (PD) or T2-weighted sequences in coronal, sagittal and axial planes.

**Table 1:** Detailed scanning parameters of our MR-arthrography protocol at 3 Tesla MRI Scanner

Sequence	Plane	Field of view in mm	Echo time in ms	Repetition time in ms	Flip angle in °
T1 TSE	Transversal	180	24	742	120
T1 TSE	Coronal	160	12	550	135
PD TSE FS	Coronal	160	33	4540	150
PD TSE FS	Sagittal	160	33	4600	150
T2 TRUFI ISO	Tansversal oblique	160	5.85	11.70	30

*mm* millimeters, *ms* milliseconds, *TSE* turbo spin-echo, *FS* fat-suppressed, *PD* proton density, *TRUFI* true fast imaging with steady-state free precession

### Evaluation of eligible MRI examinations and radiographs

All MRI examinations were independently reviewed by two readers (T.F., a fellowship-trained musculoskeletal radiologist with 8 years of experience in radiology and two years in musculoskeletal radiology; D.V., a radiology resident with 3 years of experience in general radiology) regarding the presence of SAF and classification of the subtypes. SAF was characterized as a well-circumscribed bony fossa in the acetabular roof, at least seen in two imaging planes. According to Dietrich et al. [8], type 1 was characterized as a fluid-filled (synovial fluid or contrast agency) and type 2 as bony fossa with cartilage filling. Disagreements between both readers were solved by a third, senior radiologist (T.J.D., 10 years of experience in musculoskeletal radiology).

**Fig. 1:**
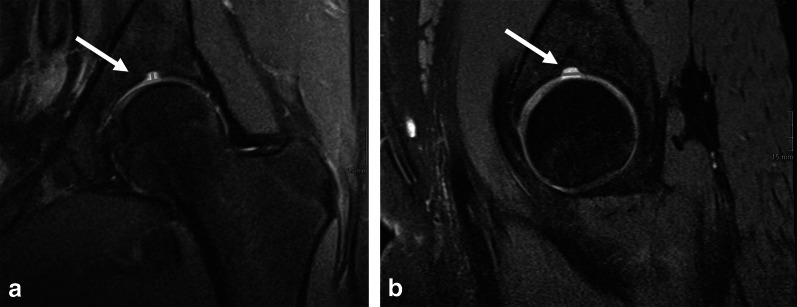
An example of the placed calipers to perform the measurements on MRI without contrast of the left hip of a 16-years-old, female participant. Proton density weighted (PD) coronal (**a**) with calipers to measure height and width (**a**), PD sagittal (**b**) with calipers to measure depth (**b**). Dimensions were 6 mm (height), 3 mm (width) mm and 7 mm (depth) (white arrows)

The SAF was measured in three dimensions, using a picture archiving and communication system (PACS) toolbox (IMPAX, Agfa HealthCare NV, Belgium); height and width in coronal planes and depth in sagittal planes. An example of the placed calipers to perform the measurements is given in Fig. 1. In a third round, the digital database was searched for plain hip radiography of included participants that were acquired with a maximum of 180 days before and after the MRI was acquired. These radiographs were blinded and were also reviewed by the same two readers and consensus read by the same senior radiologist regarding the presence of SAF.

### Statistics

Analyses were performed by a statistician (N.G.). All analyses were performed in the R programming language (version 4.0.2) [13]. The package "tableone" [14] was used to compute descriptive statistics. The package "irr" [15] was used to compute Cohen's kappa, and the package "boot" [16] was used to get the 95% confidence intervals for Cohen's kappa. The package "Hmisc" [17] was used to compute the confidence intervals for sensitivity and specificity. The package "rms" [18] was used to add restricted cubic splines to the logistic regression models.

Missing values for the size of SAF were not imputed, and thus, an available data analysis was performed. Categorical data were summarized with absolute and relative frequencies. Cohen's kappa and 95% bootstrap percentile confidence intervals with 1000 replicates were calculated as a measure of reliability. Sensitivity and specificity were calculated with corresponding exact 95% confidence intervals for binomial probabilities. Logistic regression models were calculated to estimate the influence of age on the presence of SAF type 1 and SAF type 2. For cases with more than one measurement, the participant-wise presence of SAF was defined as follows: For participants with no SAF, the age at the first measurement was incorporated in the analysis; when participants presented with SAF at some point in time, the age at the first measurement with SAF was incorporated in the analysis. The number of MRIs (1 vs > 1) was included in the models if it improved the fit. Where no linear relationship between age and the logit of the outcome was present, the linearity assumption was relaxed by adding restricted cubic splines with three knots. Influential values were inspected via plotting Cook's distance. Likewise, linear regression models were calculated to estimate the influence of age on the dimension of the supraacetabular fossa. For participants with measurements at the right and the left side, the mean of both sides was calculated. There were three participants for whom there were multiple measurements in time. These measurements were treated as if they came from different participants, thus, the correlated nature of the data was not taken into account. However, as the fraction of dependent data was tiny, it is unlikely that they have affected the results. Assumptions of linearity, normality and homoscedasticity were checked with residua.

## Results

A total of 323 participants were evaluated. There were 250 MRIs of only one side, 53 MRIs of both left and right side, and 19 MRIs repeated in time, totaling 401 MRIs.

Radiographs were found for 191 of the participants. Mean age at first MRI was 18 years (SD 4.6) with a range of 4–24 years.

A total of 63 (19.5%, median age: 16 years) and 51 (15.8%, median age 21 years) participants demonstrated a supraacetabular fossa type 1 and type 2, respectively (one participant revealed both; supraacetabular fossa type 1 on the one side and type 2 on the other side). Overall, there were 115 (35.6%) participants with and 208 (64.4%) without a SAF. Key demographics and MRI findings are given in Table 2.

**Table 2:** Key demographics, MRI-studies and findings

Variable	Overall
*n*	323
age span	4–24
Age (mean SD)	18.0 (4.6)
Male/female	186/155
MRI (%)	
Left and right	53 (16.4)
Left and right, multiple measurements	1 (0.3)
One side only	250 (77.4)
One side only, multiple measurements	19 (5.9)
SAF (%)	
No SAF	208 (64.4)
SAF	115 (35.6)
Type 1	63 (19.5)
Type 2	51 (15.8)
Both	1 (0.3)

The interrater reliability was slightly higher for MR-arthrography than for conventional MRI, which could be an indicator of higher accuracy on MR-arthrography images. Cohen's kappa for conventional MRI: 0.69 (95% CI: 0.58–0.78) Cohen's kappa for MR-arthrography images: 0.75 (95% CI: 0.66–0.83).

**Fig. 2:**
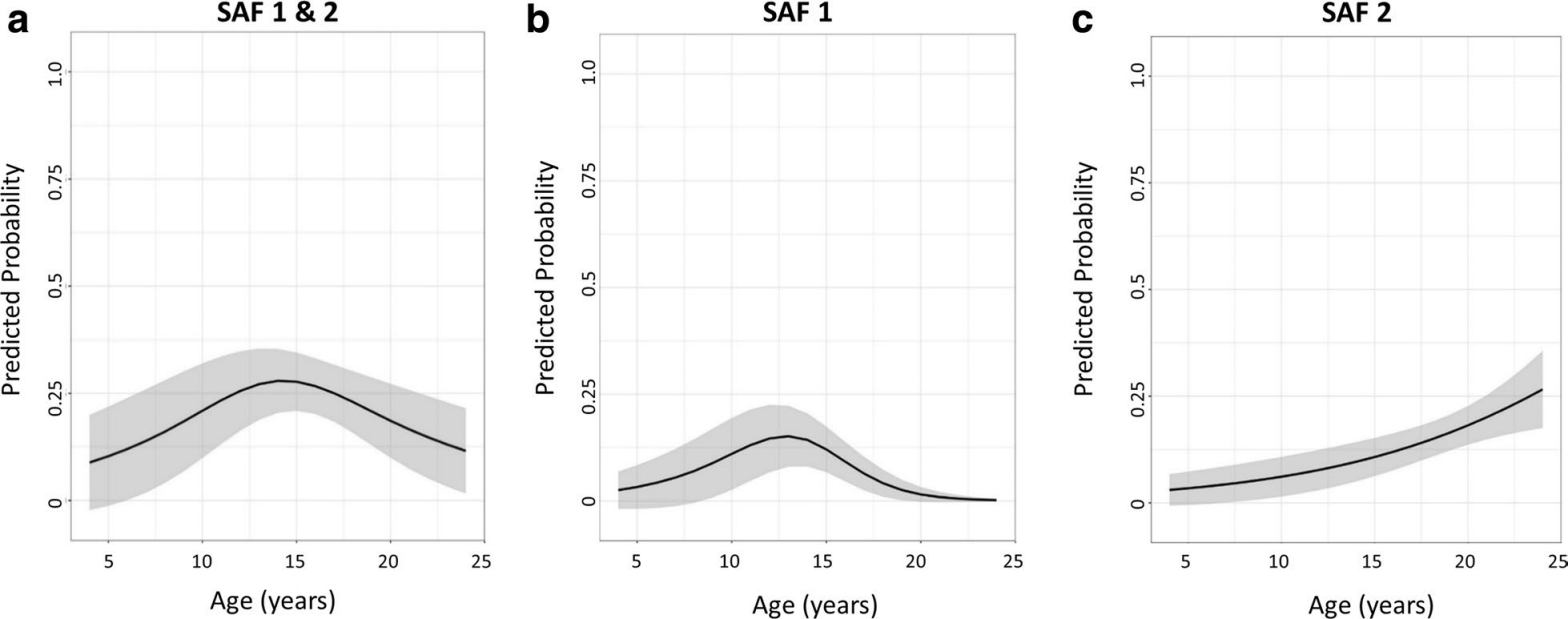
Age-dependent predicted probability with corresponding 95% confidence bands of SAF type 1/type 2 (**a**), SAF type 1 (**b**) and SAF type 2 (**c**)

Changes with age were as follows: Regardless of SAF type, the predicted probability for SAF first increased until the age of 14 before it decreased again (Fig. 2a). The predicted probability for SAF type 1 first increased until the age of 13 before it decreased again (Fig. 2b). For SAF type 2, where the logit of the probability was linearly related to age, the OR was 1.13, meaning that with each year the predicted probability for SAF type 2 increased by 13.0% (*p* = 0.002) (Fig. 2c). The size of the SAF appeared to decrease with age, i.e., the width of the SAF decreased by an average of 0.2 mm each year (*p* < 0.001), the height decreased by an average of 0.1 mm each year (*p* = 0.037), and the depth decreased by an average of 0.3 mm each year (*p* < 0.001).

**Fig. 3:**
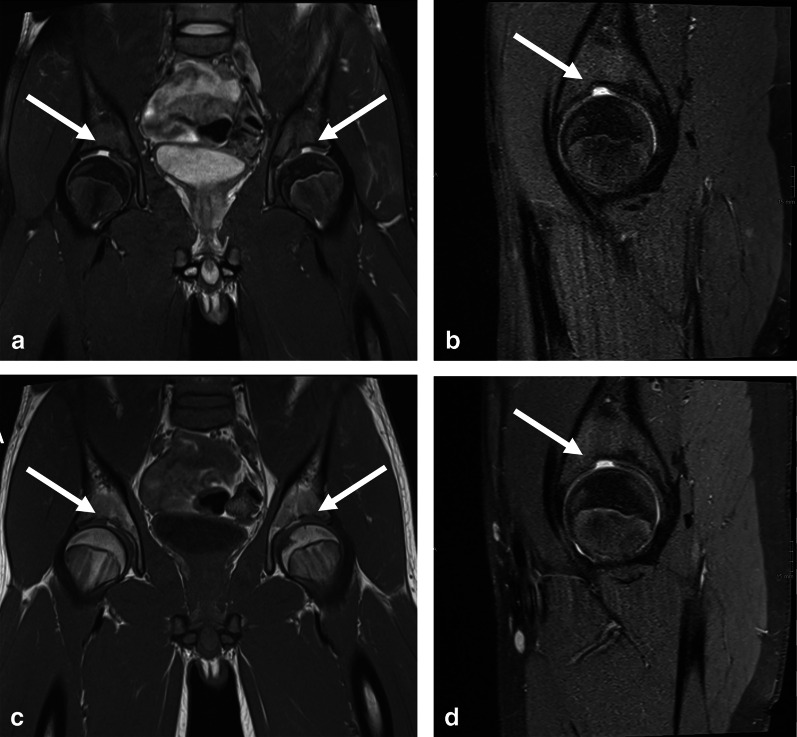
Hip-MRI of a 15-years-old, male participant without contrast with bilateral SAF type 1 (white arrow): Proton density weighted (PD) coronal both sides (**a**), PD sagittal right side (**b**), T1 weighted coronal both sides (**c**) and PD weighted sagittal left side (**d**)

**Fig. 4:**
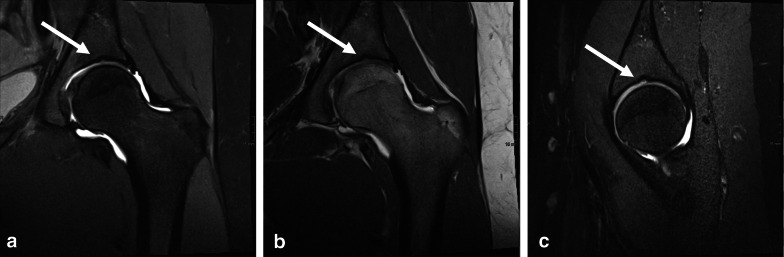
MR-Arthrography of the hip of a 21-years-old, female participant with SAF type 2 on left side (white arrow): Proton density weighted (PD) coronal (**a**), T1 weighted sagittal (**b**) and PD weighted sagittal (**c**)

**Fig. 5.**
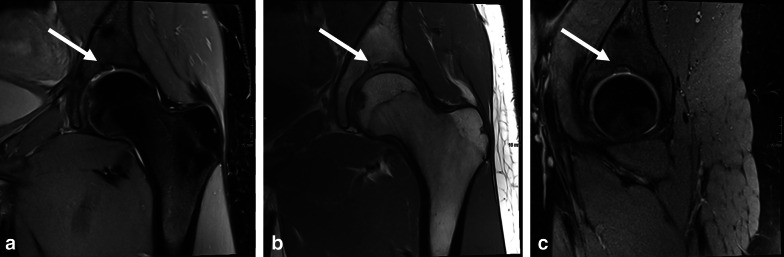
13-years-old female participant with SAF which is partly cartilage filled (white arrow). This example may represent a transitional state from SAF type 1 to SAF type 2: Proton density (PD) weighted (**a**), T1 weighted coronal (**b**) and PD weighted sagittal (**c**)

Out of 53 participants with MRIs of both sides, 12 (22.6%) and 7 (13.2%) had SAF type 1 and type 2, respectively, on both sides, and 8 (15.1%) and 1 (1.9%) had SAF type 1 and type 2, respectively, on only one side. Examples of SAF type 1 and SAF type 2 are given in Figs. 3 and 4. An example of an indetermined SAF that demonstrates type 1 and type 2 characteristics at the same time is given in Fig. 5 and may represent a transitional stage from type 1 to type 2.

Regarding evaluation of predictive factors in younger participants, we included the sex in the statistic models with SAF total, SAF type1 and SAF type 2 as an outcome. However, including sex as a predictor did not improve the fit of the models. Thus, sex was not a significant predictor of SAF.

**Fig. 6:**
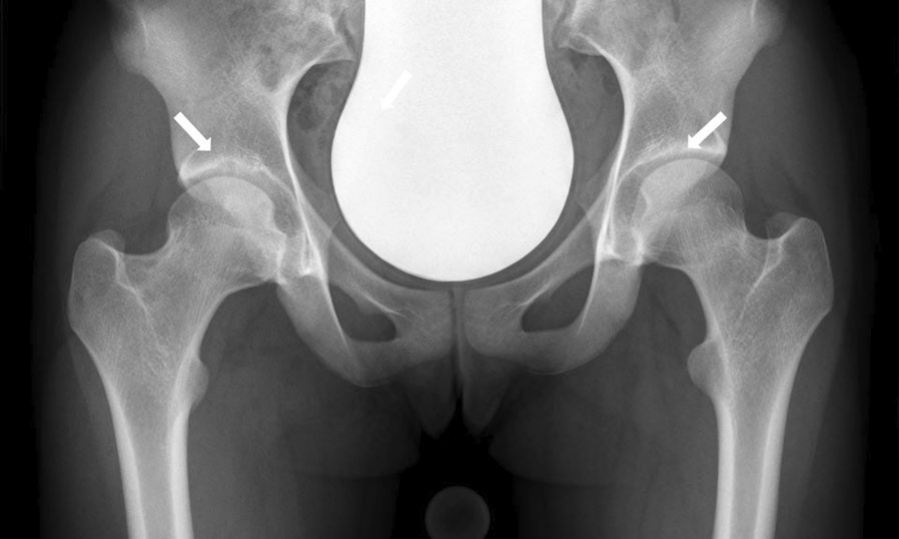
Radiograph of a 17-years-old female participant with SAF on both sides (white arrow)

Comparing radiographic and MRI diagnoses (*n* = 156) revealed a sensitivity of 0.35 (95% CI: 0.22–0.50) and a specificity of 0.93 (95% CI: 0.87–0.97). A radiograph with a SAF on both sides is given in Fig. 6.

## Discussion

This study showed that the prevalence and the size of the supraacetabular fossa are age-dependent in a young study population. The supraacetabular fossa was most frequently observed in adolescents between 12 and 17 years of age. Prevalence and size decreased with older age which suggests that the SAF is a developmental variant.

The SAF is a variant and pseudodefect of the cartilage at the acetabular roof in the 12 o'clock position and may be misinterpreted false-positive as pathology [1, 7–9], for example, a cartilage defect after septic arthritis, osteomyelitis or fracture of the pelvis with involvement of the acetabulum. In this young study cohort up to 25 years of age, the SAF was more prevalent compared to the data by Boutris et al. and Dietrich et al. in an adult population (35.6 vs 10.5%—12.6%) [8, 9]. Peak prevalence of SAF (type 1 and type 2 altogether) was seen in 14-years-old adolescents, the fluid-filled SAF type 1 was most prevalent in the 13-years-old before it decreased again. The prevalence of the cartilage-filled SAF 2 increased related to age up to 25-year-old adults in the present study population. Likewise, participants with a SAF type 1 tended to be younger compared to participants with a SAF type 2. The present data suggest that the SAF type 1 in earlier life may maturate to a cartilage-filled SAF type 2 in the older cohort of this study and may finally obliterate. Progredient cartilagefilling of the bony fossa during maturation is also supported by size measurements: the size of the SAF in all measured planes decreased significantly with increasing participant age. SAF obliteration may arrest at any time with a subsequent lifelong developmental variant in a typical location.

A similar development has been proposed for the glenoid bare spot, a cartilage pseudodefect at the shoulder, by Djebbar et al. 2018 in a retrospective study of shoulder MRIs in a participant population between 5 and 25 years [19]. Results by Djebbar et al. showed a significantly higher incidence of the glenoid bare spot in the pediatric population compared to an adult population, with a peak prevalence around 14 to 15 years. The size of the glenoid bare spot was significantly larger in the younger age groups. The authors concluded that the glenoid bare spot is a normal pattern of ossification of the glenoid surface [19]. Kim et al. [20] evaluated a population between 0 and 20 years and found the a higher prevalence of the glenoid bare spot in the age group between 10 and 20 years (2.1%) compared to 0 and 10 years (0%). In contrast to Djebbar et al., the authors concluded that the glenoid bare spot was an acquired lesion due to the even higher prevalence in an adult population (up to 80%, anatomical studies).

Sensitivity of radiographs for detection of the SAF differed to previous reports: 0.35 versus 0.68 [9]. In contrast to our study, blinding the observers for MRI findings when evaluating for a SAF was not mentioned by Boutris et al., if not done, this may be an explanation for the difference in the results of the present article. Specificity was not evaluated by Boutris et al.

While performing our study, we did not notice any predictive factors in younger participants or predictive factors of persistence into adulthood. This may be an interesting aspect for further studies.

Limitations of the study are related to the retrospective design which leads to more statistical uncertainty compared to a prospective design with fixed follow-up intervals. The interrater reliability was slightly higher on MR- arthrography images than for conventional MRI, which could be an indicator of higher accuracy on MR-arthrography images. Moreover, while conventional MRI was used for all ages, MR-arthrography was used only for children aged 12 years or older. Thus, diagnostic accuracy might have been higher for older than for younger children.

In conclusion, the supraacetabular fossa is most frequent in adolescents. The data of the present study suggest that the SAF is a developmental variant. During skeletal maturation, the SAF may evolve from type 1 as fluid-filled fossa to a cartilage-filled type 2 to no fossa. Evolution may arrest at any stage.

## Data Availability

The dataset analyzed during this study are available from the corresponding author on reasonable request.

## Abbreviations

FS: Fat-suppressed
MRI: Magnetic resonance imaging
PACS: Picture archiving and communication system
PD: Proton density
SAF: Supraacetabular fossa
SD: Standard deviation
TRUFI: True fast imaging with steady-state free precession
TSE: Turbo spin-echo
